# Timing of inspiratory muscle activity detected from airway pressure and flow during pressure support ventilation: the waveform method

**DOI:** 10.1186/s13054-022-03895-4

**Published:** 2022-01-30

**Authors:** Francesco Mojoli, Marco Pozzi, Anita Orlando, Isabella M. Bianchi, Eric Arisi, Giorgio A. Iotti, Antonio Braschi, Laurent Brochard

**Affiliations:** 1 Anesthesia and Intensive Care, Emergency Department, Fondazione IRCCS Policlinico S. Matteo, viale Golgi 19, 27100 Pavia, Italy; 2 Anesthesia, Intensive Care and Pain Therapy, Department of Clinical-Surgical, Diagnostic and Pediatric Sciences, University of Pavia, Pavia, Italy; 3 Keenan Research Centre, Li Ka Shing Knowledge Institute, St. Michael’s Hospital, Toronto, Canada; 4 Interdepartmental Division of Critical Care Medicine, University of Toronto, Toronto, Canada

**Keywords:** Pressure support ventilation, Spontaneous respiratory activity, Respiratory effort, Ventilator waveforms, Patient–ventilator interaction, Asynchronies

## Abstract

**Background:**

Whether respiratory efforts and their timing can be reliably detected during pressure support ventilation using standard ventilator waveforms is unclear. This would give the opportunity to assess and improve patient–ventilator interaction without the need of special equipment.

**Methods:**

In 16 patients under invasive pressure support ventilation, flow and pressure waveforms were obtained from proximal sensors and analyzed by three trained physicians and one resident to assess patient’s spontaneous activity. A systematic method (the waveform method) based on explicit rules was adopted. Esophageal pressure tracings were analyzed independently and used as reference. Breaths were classified as assisted or auto-triggered, double-triggered or ineffective. For assisted breaths, trigger delay, early and late cycling (minor asynchronies) were diagnosed. The percentage of breaths with major asynchronies (asynchrony index) and total asynchrony time were computed.

**Results:**

Out of 4426 analyzed breaths, 94.1% (70.4–99.4) were assisted, 0.0% (0.0–0.2) auto-triggered and 5.8% (0.4–29.6) ineffective. Asynchrony index was 5.9% (0.6–29.6). Total asynchrony time represented 22.4% (16.3–30.1) of recording time and was mainly due to minor asynchronies. Applying the waveform method resulted in an inter-operator agreement of 0.99 (0.98–0.99); 99.5% of efforts were detected on waveforms and agreement with the reference in detecting major asynchronies was 0.99 (0.98–0.99). Timing of respiratory efforts was accurately detected on waveforms: AUC for trigger delay, cycling delay and early cycling was 0.865 (0.853–0.876), 0.903 (0.892–0.914) and 0.983 (0.970–0.991), respectively.

**Conclusions:**

Ventilator waveforms can be used alone to reliably assess patient’s spontaneous activity and patient–ventilator interaction provided that a systematic method is adopted.

**Supplementary Information:**

The online version contains supplementary material available at 10.1186/s13054-022-03895-4.

## Background

During mechanical ventilation patient–ventilator asynchrony is frequent and is associated with unfavorable outcomes [1–4]. Poor patient–ventilator interaction can be a marker of disease severity or result from inappropriate settings; it may be a cause of direct damage or can indirectly affect outcome through inappropriate sedation or undue prolongation of weaning [2, 4–9]. Asynchronies have different underlying mechanisms and may have different impacts on outcome as well. Thus, phenotyping patients according to their interaction with the ventilator can be important to identify patients at risk and guide treatments.

Ventilator waveform interpretation was originally described in the 90 s’ to assess patient–ventilator interaction [5, 10] and it was proposed as a skill that intensivists should possess in the era of mechanical ventilators displaying real-time waveforms [11]. Detection of major asynchronies (i.e., ineffective efforts, auto-triggered and double-triggered breaths) was found to be highly reproducible and reliable when using waveform recordings [2]. A few years later, however, another study showed a low sensitivity in detecting the same events when waveforms displayed by the ventilator were compared to electrical activity of the diaphragm [12]. It has then been suggested that specific training focused on ventilator waveform interpretation is needed to detect major asynchronies at the bedside [13]. In addition, no data are available about waveform detection of minor asynchronies such as trigger delay, early and late cycling. These “minor” asynchronies predispose to and are more frequent than “major” asynchronies [5, 8, 14, 15], accounting for more than 75% of the total asynchrony time in patients under pressure support ventilation [16]. Prerequisite for the detection of minor asynchronies is the ability to precisely identify the start and the end of patient’s spontaneous respiratory effort.

We designed a systematic and explicit method (the waveform method), based on simple specific rules based on respiratory physiology, to detect the activity of patient’s respiratory muscles from ventilator waveforms under pressure support ventilation. The aim of this study is to test the hypothesis that the waveform method is reliable and reproducible in providing a precise assessment of the timing of the inspiratory muscles’ effort. This could have a great potential for an automated real-time analysis of ventilator waveforms, to monitor synchrony at the bedside, to directly guide the ventilator triggering and cycling functions, to prevent asynchronies and improve patient–ventilator interaction during pressure support ventilation.

## Materials and methods

In this prospective observational study, we enrolled mechanically ventilated patients in pressure support ventilation (PSV) mode with an esophageal balloon already inserted for clinical purposes, either because they displayed any form of asynchrony on the ventilator screen visible at the bedside or they were considered by clinicians to be uncomfortable from a ventilation standpoint. Tachypnea, activation of accessory muscles, abdominal paradox, diaphoresis, and/or agitation were considered clinical signs of respiratory discomfort. The study was approved by the ethics committee of our institution (Fondazione IRCCS Policlinico San Matteo, n. 41223) and written informed consent to use the recordings for research purposes was obtained from all patients as soon as they were able to provide it.

### Recordings

Patients were connected to a Hamilton Medical G5 ventilator (Bonaduz, Switzerland) or a GE Healthcare Engstrom (Madison (WI), USA) ventilator, both equipped with proximal pressure and flow sensors (i.e., located at the Y-piece of the respiratory circuit) and an auxiliary port for esophageal balloon catheter (NutriVent catheter; Sidam, Mirandola, Italy) connection. Airway pressure (Paw), flow and esophageal pressure (Pes) tracings were recorded at 100 Hz for approximately 10 min.

To better describe the population studied, the expiratory time constant was computed from the tracings as the slope of expiratory flow/volume relationship during passive expiration [17]. Respiratory mechanics were then defined as being restrictive, normal or obstructive according to the expiratory time constant < 0.4 s, 0.4–0.7 s, > 0.7 s, respectively.

## Reference method: esophageal pressure tracings to detect patient respiratory efforts

**Fig. 1:**
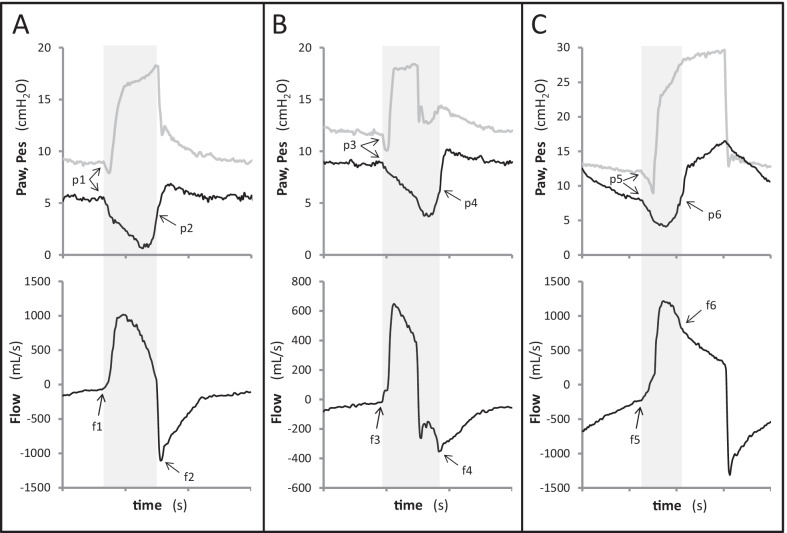
Waveform detection of patient respiratory activity and minor asynchronies. Good synchronization is shown in **A** while minor asynchronies are displayed in **B** (cycling is too early) and **C** (trigger is delayed and cycling is late). Airway (Paw, gray line) and esophageal (Pes, black line) pressures are displayed on the top and flow at the bottom. Gray-colored areas refer to subject’s neural inspiratory time according to Pes tracing. The start of patient’s inspiration can be detected as a negative deflection on both Paw and Pes (p1, p3 and p5) and as a positive deflection of flow (f1, f3 and f5). Normally the end of patient’s inspiration occurs at mid-relaxation of the inspiratory muscles and can be located at midpoint of the fast increase in Pes after its inspiratory nadir (p2, p4 and p6); this time point corresponds also to the start of a phase of exponential decay of flow (f2, f4 and f6). Substantial deviations from normal exponential decay of expiratory flow are associated with early cycling (**B**) and inspiratory trigger delay (**C**). Prolonged exponential decay of inspiratory flow is associated with a secondary phase of passive inflation due to cycling delay (**C**)

**Fig. 2:**
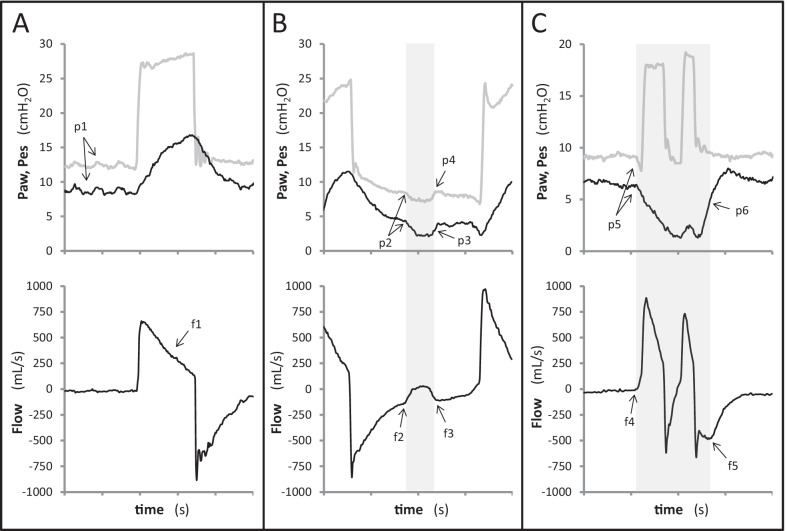
Waveform detection of patient respiratory activity and major asynchronies. Airway (Paw, gray line) and esophageal (Pes, black line) pressures are displayed on the top and flow at the bottom. Gray-colored areas refer to the subject’s neural inspiratory time according to Pes tracing. The breath in **A** is an autotriggered mechanical breath favored by heart noises that are evident both in airway and esophageal pressure tracings (p1). Conversely, no clear sign of patient’s inspiratory effort can be detected on flow, airway and esophageal pressure right before the mechanical breath; inspiratory flow shows exponential decay from its peak value, suggesting passive inflation (f1). In **B**, gray-colored area marks a patient’s inspiratory effort that is not recognized nor assisted by the ventilator. The start of patient’s inspiration can be detected as a negative deflection on Paw and Pes (p2) and a positive deflection of flow (f2) that interrupts the normal exponential decay of passive expiratory flow. The end of patient’s inspiration is located at mid-relaxation of inspiratory muscles (p3) and can be detected as the re-start of the normal exponential decay of expiratory flow (f3) and the end of a negative deflection of Paw (p4). In **C**, gray-colored area marks a single patient’s inspiratory effort that triggers two distinct mechanical breaths, separated by a brief expiratory phase (double trigger). The start of patient’s inspiration can be detected as a negative deflection on Paw and Pes (p5) and a positive deflection of flow (f4). The end of patient’s effort is located at mid-relaxation of inspiratory muscles (p6), occurs well after the cycling of the second mechanical breath and corresponds to the start of a normal, exponentially decaying expiratory flow (f5)

Esophageal pressure was used as the reference in the assessment of patient inspiratory activity [18, 19]. The start of patient’s inspiratory effort (patient Ti-start) was detected as a sudden negative deflection of Pes (Figs. 1,2).

The end of inspiration was determined as follows. In normal unassisted breathing, inspiratory to expiratory flow reversal occurs before full relaxation, when the outward pressure still generated by the inspiratory muscles is exactly counterbalanced by the inward pressure generated by the elastic recoil of the respiratory system [20]. The exact time point of flow reversal depends on respiratory mechanics and breathing pattern, but can be approximated at mid-relaxation of the inspiratory muscles [21, 22]. In the absence of a universal definition [23], the end of patient’s inspiration (patient Ti-end) was pragmatically located at midpoint of the fast increase in Pes after its inspiratory nadir (Figs. 1,2).

### Waveform method to detect patient’s respiratory effort

The Waveform method is based on the analysis of flow and airway pressure, according to five general physiological principles displayed in Table 1 and to the specific rules detailed below [5, 10, 11, 22].

**Table 1:** General principles of the waveform method for detection of patient spontaneous efforts and assessment of patient–ventilator interaction during pressure support ventilation

General principles of the waveform method under pressure support ventilation
1. Normal “physiologic” breathing pattern is made of active inspiration and passive expiration
2. An exponential decay of flow suggests passive condition: this is valid both for inspiratory and expiratory flow
3. Ideally, during synchronous pressure support perfectly matching subject’s inspiratory effort, passive conditions are not observed during the ventilator inspiratory phase, whereas the ventilator expiratory phase reflects passive conditions
4. During the ventilator inspiratory phase, the presence of passive conditions indicates auto-triggering or delayed cycling
5. During the ventilator expiratory phase, deviation from passive conditions indicates trigger delay, ineffective efforts, early cycling or expiratory muscle activation

The start of a subject’s inspiratory effort (wave Ti-start) is detected on flow, as a sudden positive deflection that interrupts a phase of exponential decay, and/or on airway pressure, as a sudden negative deflection that interrupts a phase of stable Paw (Figs. 1,2).

For the end of a subject’s inspiration (wave Ti-end) it is assumed that the interruption of an inspiratory effort is followed by a phase of exponential decay of flow. Therefore, the end of the patient’s inspiratory effort is pinpointed at the start of a passive exponential decay of flow, irrespective of the direction of flow (inspiratory or expiratory). Examples are provided in Figs. 1 and 2. If cycling is optimal, the peak expiratory flow is immediately followed by an exponential decay (Fig. 1A). If cycling occurs early, a delayed start of the exponential decay of expiratory flow marks the end of the inspiratory effort occurring during expiration (Fig. 1B). If cycling occurs late, an exponential decay occurs during the inspiratory flow period after a change in slope (Fig. 1C). A pure exponential decay during the whole inspiration indicates auto-triggering (Fig. 2A). In case of ineffective effort, the normal exponential decay of expiratory flow is interrupted (Fig. 2B). The end of a negative deflection of Paw can also be associated with the end of a subject’s inspiration (Fig. 1B).

## Reference assessment of patient–ventilator interaction

The start of the mechanical inspiratory and expiratory phases (machine Ti-start and machine Ti-end) were detected on Paw as the start of pressurization and depressurization. According to mechanical and patients’ reference times, breaths were defined as assisted, auto-triggered, double-triggered (Fig. 2C) or ineffective. In assisted breaths, machine ΔTi-start and ΔTi-end were computed as machine Ti-start minus patient Ti-start and machine Ti-end minus patient Ti-end. The time gap between the patient and the ventilator was considered substantial when > 250 ms, corresponding to > 25% of a normal inspiratory time. Thus, trigger delay was defined as machine ΔTi-start > 250 ms; early and late cycling were defined as machine ΔTi-end < − 250 ms and > 250 ms respectively. Breaths in which machine ΔTi-start was < 250 ms, breaths in which machine ΔTi-end (absolute value) was < 250 ms, and breaths in which both machine ΔTi-start and ΔTi-end (absolute values) were < 250 ms were counted.

Asynchrony index was the percentage of breaths affected by major asynchronies and was computed as the sum of auto-triggered, ineffective and double-triggered breaths, divided by the total number of breaths. Total asynchrony time was the time (expressed as percentage of total recording time) during which the ventilator and the patient were not synchronous; it was computed as the sum of machine ΔTi-start and ΔTi-end (absolute value) in assisted breaths plus the time length of auto-triggered breaths and of ineffective efforts.

### Waveform method performance

Each patient’s recording was analyzed by a “reference” operator (provided with both the standard waveforms and Pes tracing) and by another “waveform” operator (blinded to Pes), in order to assess the agreement between the reference and the waveform method. Additionally, a random selection of 30 min (approximately 120 s and 30 breaths per patient) was analyzed by three operators, in order to assess the inter-operator agreement of the waveform method (details are provided in Additional file 1: Table S1). All the operators (three senior physicians and one resident) underwent previous specific training and had experience (at least 2 years) of waveform interpretation for both clinical and research purposes. To assess the reliability of the waveform method, detected breaths (efforts detected on Pes and on waveforms), false positive breaths (efforts detected on waveforms but not on Pes) and false negative breaths (efforts detected on Pes but not on waveforms) were counted. To assess the precision in detecting the start and the end of the patient effort, wave ΔTi-start (wave Ti-start minus patient Ti-start) and wave ΔTi-end (wave Ti-end minus patient Ti-end) were computed; moreover, breaths in which wave ΔTi-start was < 250 ms, breaths in which wave ΔTi-end (absolute value) was < 250 ms, and breaths in which both wave ΔTi-start and wave ΔTi-end (absolute values) were < 250 ms were counted.

Similar to the reference analysis, wave Ti-start and wave Ti-end were used to assess patient–ventilator interaction with the waveform method and the findings compared with the reference.

### Statistical analysis

The primary endpoint of the study was the percentage of spontaneous efforts detected by the waveform method. To get a confidence interval of ± 2% with a confidence level of 99%, a sample of 4160 breaths was required. Secondary endpoints included the percentage of detected efforts in which both wave ΔTi-start and wave ΔTi-end were < 250 ms (absolute values), the agreement between the waveform method and the reference in detecting major and minor asynchronies, and the inter-rater agreement when the method was applied by different physicians. Agreement of the waveform method with the reference in rating breaths as assisted, auto-triggered, double-triggered or ineffective was assessed with Cohen’s kappa. Sensitivity, specificity, area under the curve, positive and negative predictive values in detection of trigger delay, early and late cycling were computed. Asynchrony times assessed with the waveform method were compared to the reference with paired Wilcoxon test. Agreement among raters was assessed with intraclass correlation coefficient for single measures. Categorical variables are displayed as absolute number and percentage, continuous variables are displayed as mean value ± standard deviation or median value and interquartile range (IQR), as appropriate.

## Results

We analyzed 4426 breaths that were recorded (total recording time 174 min) in sixteen patients: 7 males and 9 females, 55 (IQR 43 to 63] years old, under pressure support ventilation with evidence of suboptimal interaction with the mechanical ventilator, as defined in methods. Causes of respiratory failure were ARDS (2), pneumonia (2), lung fibrosis (1), COPD decompensation (3), congestive heart failure (2), postoperative (3), sepsis (2) and pancreatitis (1). According to the expiratory time constant, 4 patients displayed a restrictive flow pattern, 5 normal and 7 obstructive. Patients’ characteristics are summarized in Additional file 1:Table S2.

### Breaths and delays: machine performance

**Fig. 3:**
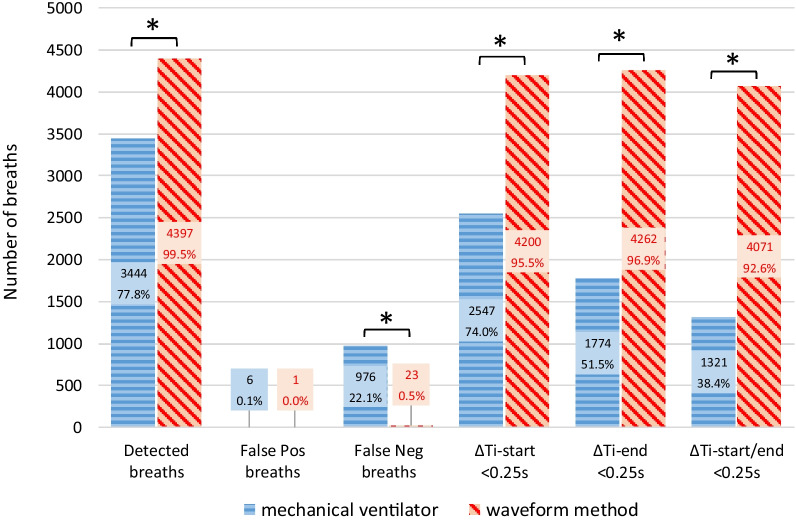
Performance of the mechanical ventilator versus the waveform method in detecting patient inspiratory efforts. Compared to the mechanical ventilator (blue bars), the waveform method (red bars) detected substantially more patient efforts. Among detected breaths, the proportion of those without minor asynchronies (ΔTi-start and/or ΔTi-end < 0.25 s) was larger for the waveform method. For detected, false positive and false negative breaths, % values refer to the total number of breaths (N = 4426); for breaths with ΔTi-start and/or ΔTi-end < 0.25 s, % values refer to the number of detected breaths (N = 3444 for the machine, N = 4397 for the waveform method). *p < 0.0001 mechanical ventilator versus waveform method.

Out of 4426 total breaths, 3444 (77.8%) were detected and assisted, 976 (22.1%) were not detected by the ventilator (ineffective efforts) and 6 (0.1%) were auto-triggered; no double-triggered breaths were observed. In assisted breaths, machine ΔTi-start was 200 ± 139 ms ranging 0 to 1002 ms and machine ΔTi-end was 195 ± 361 ms ranging − 1023 to 2258 ms. Trigger delay was detected in 897 (26.0%) assisted breaths, late cycling occurred in 1231 (35.7%) and early cycling in 439 (12.7%). Machine ΔTi-start and ΔTi-end were < 250 ms in 2547 (74.0%) and 1774 (51.5%) assisted breaths respectively (Fig. 3); breaths that were properly assisted without minor asynchronies were 1321 (38.4%). Early cycling was detected only in the 4 restrictive patients, whereas late cycling was detected in all obstructive and normal patients and in 2 restrictive patients (Additional file 1:Table S3).

The Asynchrony Index was 5.9% (IQR 0.6–29.6) ranging 0.0–45.5. The total asynchrony time represented 22.4% (16.3–30.1) of total recording time, ranging 11.3–47.3 (Additional file 1: Figure S1). Minor asynchronies lasted more than major ones (*p* = 0.0013): 16.5% (14.0–20.4) of total recording time, accounting for 92.1% (66.2–99.2) of total asynchrony time.

### Performance of the waveform method

Among 4420 spontaneous efforts identified on the esophageal pressure tracings, 4397 (99.5%) were also detected by the expert waveform analysis using only airway pressure and flow. False negative (not detected) breaths were 23 (0.5%; *p* < 0.0001 vs. machine) and only 1 (0.0%) false positive breath was observed (Fig. 3). For breaths detected on waveforms, wave ΔTi-start was − 7 ± 106 ms (*p* < 0.0001 vs. machine ΔTi-start) and wave ΔTi-end was − 21 ± 96 ms (*p* < 0.0001 vs. machine ΔTi-end). Overall, breaths with wave ΔTi-start < 250 ms were 4200 (95.5%; *p* < 0.0001 vs. machine), breaths with wave ΔTi-end < 250 ms were 4262 (96.9%; *p* < 0.0001 vs. machine) and breaths with both wave ΔTi-start and ΔTi-end < 250 ms were 4071 (92.6%; *p* < 0.0001 vs. machine). Findings in individual patients are displayed in Additional file 1: Table S4. The global agreement between the reference and the waveform methods in defining breaths as assisted, auto-triggered, double-triggered or ineffective was excellent, as assessed by a Cohen’s Kappa of 0.98 (0.98 – 0.99). Out of 4426 total breaths (4420 patients efforts and 6 autotriggers), 17 ineffective efforts were undetected, 6 assisted breaths were erroneously considered autotriggers and 1 autotrigger was erroneously considered an assisted breath by the waveform method (Additional file 1: Table S5). The Asynchrony Index did not differ when assessed with the waveform method versus esophageal pressure.

Sensitivity, specificity, positive and negative predictive values of the waveform method in detecting asynchronies are reported in Table 2. Area under the curve (95% confidence interval; *p* value) for trigger delay, cycling delay and early cycling was 0.865 (0.853–0.876; *p* < 0.0001), 0.903 (0.892–0.914; *p* < 0.0001) and 0.983 (0.970–0.991; *p* < 0.0001) respectively (Additional file 1: Figure S2).

**Table 2:** Performance of the waveform method in detection of major and minor asynchronies

	Sensitivity (%)	Specificity (%)	PPV (%)	NPV (%)
Assisted breath	99.8 (99.6–99.9)	99.9 (99.4–100.0)	77.8 (76.6–79.0)	99.4 (98.7–99.8)
AutoTriggering	83.3 (35.9–99.6)	99.9 (99.7–100.0)	45.5 (16.7–76.6)	100.0 (99.9–100.0)
Ineffective effort	98.3 (97.2–99.0)	100.0 (99.9–100.0)	100.0 (99.6–100.0)	99.5 (99.2–99.7)
Trigger delay	76.8 (73.9–79.5)	90.0 (89.0–91.0)	66.3 (63.3–69.1)	93.9 (93.0–94.6)
Late cycling	89.1 (87.2–90.8)	83.8 (82.4–85.0)	67.9 (65.5–70.2)	95.2 (94.4–96.0)
Early Cycling	93.2 (90.4–95.3)	99.6 (99.3–99.8)	96.2 (94.0–97.8)	99.3 (98.9–99.5)

Sensitivity, specificity, positive predictive value (PPV) and negative predictive value (NPV) of waveform detection of ineffective efforts, trigger delay, early and late cycling are displayed as percentage (95% confidence interval). No double-triggered breaths were observed

Total asynchrony time and asynchrony times related to ineffective efforts, auto-triggered breaths, trigger delay, early and late cycling were not different when assessed with the Waveform method compared to the reference (Additional file 1: Figure S1).

Among the three “waveform” operators, the agreement for single measures was 0.99 (0.98 – 0.99) in classifying breaths as auto-triggered, double triggered, assisted or ineffective, 0.84 (0.76 – 0.89) in measuring ΔTi-start and 0.96 (0.90 – 0.98) in measuring ΔTi-end.

## Discussion

The main findings of this study are: (1) the waveform method allows a very precise assessment of the timing of patients’ spontaneous activity using only pressure and flow waveforms during PSV, (2) the method is highly reproducible and reliable in detecting both major and minor asynchronies, and (3) during PSV the majority of total asynchrony time is related to “minor” asynchronies.

In the present study we prospectively enrolled ICU patients under PSV with evidence of suboptimal interaction with the mechanical ventilator, as suggested by subject’s discomfort and/or asynchronies visible on the ventilator screen. In this selected population, experts were able to detect more than 99% of patients’ spontaneous efforts looking at standard ventilator waveforms, and in more than 90% of cases, both the start and the end of respiratory efforts were identified with good precision. Waveform detection of major asynchronies was almost in perfect agreement with the reference with very good agreement among different raters. The waveform method allowed a precise assessment of the timing of patients’ spontaneous effort. Thus waveform recognition of minor asynchronies is highly reliable and reproducible as well. Looking at the composition of total asynchrony time in individual patients, the picture was very similar whether the assessment was performed with the reference or the waveform method.

Once asynchronies are detected at the bedside, patient–ventilator interaction can be optimized with both general and specific measures [5–7, 10, 21, 22, 24–27]. Proper ventilator settings -including pressure support level, pressurization rate, inspiratory and expiratory trigger sensitivity- can substantially improve the interaction. Adjustments of ventilator settings must be guided by bedside assessment of asynchronies and be individualized [5, 11, 22, 24–31]. Although esophageal pressure is the reference method to assess patient’s respiratory activity and patient–ventilator interaction [18, 19], it is moderately invasive, requires special equipment and some expertise to manage technical issues, and generates additional costs as well. Because asynchronies are very frequent in mechanically ventilated patients, esophageal pressure cannot be the standard approach. Our findings suggest that the interpretation of bedside waveform, readily available, can identify patients with poor interaction with the mechanical ventilator, quantify the problem, define the specific asynchrony pattern and guide the ventilator setting.

Automatic real-time analysis of ventilator waveforms has been described to monitor and possibly improve patient–ventilator interaction [4, 32–34]. Triggering and cycling-off functions guided by waveforms were originally implemented on mechanical ventilators for noninvasive respiratory support to overcome the issue of large air leaks [35]. The performance of waveform method, designed for invasive ventilation setting and tested in intubated patients, should encourage further advancement. Automation of the method is particularly attractive having the potential to decrease asynchronies and being at the same time non-invasive, low-cost and easy to be integrated in a mechanical ventilator. Machine learning has been already applied in the field with promising results [36–39].

Our results are different from those of Colombo and colleagues, who observed good specificity but insufficient sensitivity in waveform detection of major asynchronies [12]. Three main aspects explain the different performances of waveform interpretation in the present study. First, we explicitly described a method based on general principles and specific rules that was adopted beforehand. All the raters of the present study were considered reasonably well trained in ventilator waveform analysis, whereas clinical experience in treating ICU mechanically ventilated patients is not invariably associated with this skill [13]. To note, one of our expert raters was a resident when she analyzed the recordings of the present study. Second, all mechanical ventilators were equipped with proximal sensors and this may have substantially improved the reliability of pressure and flow tracings. Third, as reference signal we used esophageal pressure instead of electrical activity of the diaphragm (EADi); differently from EADi, esophageal pressure is affected by the activity of other main and accessory respiratory muscles thus providing a more comprehensive information on patient’s spontaneous activity. Moreover, the use of EADi to guide positive-pressure ventilation has been associated with a significant increase in auto-triggered and double-triggered breaths, suggesting that EADi may sometimes lack specificity [40–42].

Total asynchrony time was computed in our patients as the sum of all the different asynchronous events. Overall, asynchronies accounted for 22% of the time on mechanical ventilation with values close to 50%. Most of this time was related to the so-called minor asynchronies, a result consistent with previous findings [5, 16]; in particular, delayed cycling is confirmed to be a major problem of PSV [43–45]. Minor asynchronies predispose to major ones: late cycling promotes dynamic hyperinflation finally leading to ineffective efforts [5, 46], whereas early cycling is a pre-requisite for double triggering [8, 24]. Different asynchronies have also different meanings and can impact differently on outcome. For instance, trigger delay, late cycling and ineffective efforts are often associated with over-assistance and/or over-sedation [2, 5, 28, 43], predisposing to diaphragm disuse atrophy, prolonged mechanical ventilation and ICU stay [2–4, 29]. Both early cycling and ineffective efforts correspond to eccentric contractions of the inspiratory muscles, a potential mechanism of muscle fiber injury [9]. Double trigger with associated breath stacking represents instead an obvious risk of lung baro- and volo-trauma [8]. For all these reasons, it might be very useful to recognize (and hopefully correct or prevent) all the different asynchronous events at the bedside.

This study has limitations. First, the study population was small, raising the question whether our findings are generalizable or not. However, our case-mix was quite representative of a general ICU population in terms of age, etiology of respiratory failure and respiratory mechanics; moreover, the number of analyzed breaths was high and exceeded the sample size requirements. Second, our experts performed an off-line analysis of ventilator waveforms recorded by proximal sensors and the results could be different if the method was applied automatically by the machine and/or proximal sensors were not available.

## Conclusions

Our findings support the notion that standard ventilator waveforms can reliably be used to accurately assess patient’s spontaneous activity and patient–ventilator interaction at the bedside, provided that a systematic method is adopted after sufficient training.

## Supplementary Information

**Additional file 1.** Patients' characteristics and waveform method performance.

## Data Availability

All data generated or analyzed during this study are included in this published article and its supplementary information files.

## Abbreviations

PSV: Pressure support ventilation
Paw: Airway pressure
Pes: Esophageal pressure
Patient Ti-start: The start of patient’s inspiratory effort detected on esophageal pressure wave
Patient Ti-end: The end of patient’s inspiratory effort detected on esophageal pressure wave
Wave Ti-start: The start of a subject’s inspiratory effort detected on flow and/or pressure waveforms
Wave Ti-end: The end of a subject’s inspiratory effort detected on flow and/or pressure waveforms
Machine Ti-start: The start of the mechanical inspiratory phase
Machine Ti-end: The end of the mechanical expiratory phase
Machine ΔTi-start: Machine Ti-start minus patient Ti-start
Machine ΔTi-end: Machine Ti-end minus patient Ti-end
Wave ΔTi-start: Wave Ti-start minus patient Ti-start
Wave ΔTi-end: Wave Ti-end minus patient Ti-end
IQR: Interquartile range
ARDS: Acute respiratory distress syndrome
COPD: Chronic obstructive pulmonary disease
ICU: Intensive care unit
EaDi: Electrical activity of the diaphragm
