# Supplementary material for: Timing of inspiratory muscle activity detected from airway pressure and flow during pressure support ventilation: the waveform method
Source: Crit Care. 2022 Jan 30;26:32. doi: 10.1186/s13054-022-03895-4 (PMC8802480; doi:10.1186/s13054-022-03895-4)
Supplement: Supplementary file 1 — Additional file 1. Patients' characteristics and waveform method performance. [file 13054_2022_3895_MOESM1_ESM.docx]

**Timing of inspiratory muscle activity detected from airway pressure and flow during pressure support ventilation. The waveform method.**

**SUPPLEMENTAL MATERIAL**

**Fig. 1S Total asynchrony time**

Total asynchrony time is expressed as percentage of total recording time and split into the different types of asynchronies. R refers to the reference method (esophageal pressure) and W refers to the waveform method. Total asynchrony time and asynchrony times related to ineffective efforts, autotriggers, trigger delay, early and late cycling were not different when assessed with the waveform method compared to the reference. Minor asynchronies lasted more than major ones (p=0.0013): 16.5 % (14.0-20.4) vs. 1.8 % (0.1-10.1) of total recording time, accounting for 92.1 % (66.2-99.2) of total asynchrony time.

**Figure 2S: Receiver operating characteristic curve for waveform detection of significant trigger delay, early and late cycling**

Significant trigger delay was defined as delta trigger > 250 ms; significant early and late cycling were defined as delta cycling < -250 ms and > 250 ms respectively. Area under the curve (AUC) for trigger delay (continuous line), early cycling (dashed line) and late cycling (dotted line) are displayed in figure.

| **Patient n°** | **Ref. Operator** | **Waves Operator** | **Selected recordings** |
| --- | --- | --- | --- |
| 1-2 | AO | IB | IB, FM, MP |
| 3-4 | AO | FM | FM, MP, IB |
| 5-6 | IB | AO | AO, FM, MP |
| 7-8 | IB | MP | MP, FM, AO |
| 9-10 | FM | MP | MP, AO, IB |
| 11-12 | FM | IB | IB, AO, MP |
| 13-14 | MP | FM | FM, AO, IB |
| 15-16 | MP | AO | AO, FM, IB |

**Table 1S: Reference and waveforms operators of the study**

Reference operators analyzed both standard waveforms and esophageal pressure tracings, thus providing the reference assessment of patient-ventilator interaction. Waveforms operators were instead blinded to esophageal pressure and provided the waveforms assessment of patient-ventilator interaction. To evaluate reproducibility of the waveform method, a 2-minute selection from each patient recording (total recording time 30 minutes, total number of breaths 544) was analyzed by 3 operators blinded to esophageal pressure. AO = Anita Orlando; FM = Francesco Mojoli; IB = Isabella Bianchi; MP = Marco Pozzi.

| **Patient** | **Flow pattern** | **Male gender** | **Age (yo)** | **etiology** |
| --- | --- | --- | --- | --- |
| P1 | Restrictive | 0 | 54 | ARDS |
| P2 | Restrictive | 1 | 41 | Pancreatitis |
| P3 | Restrictive | 0 | 51 | Lung fibrosis |
| P4 | Restrictive | 1 | 35 | ARDS |
| P5 | Normal | 0 | 43 | Sepsis |
| P6 | Normal | 0 | 59 | Postoperative |
| P7 | Normal | 0 | 45 | Pneumonia |
| P8 | Normal | 0 | 41 | Sepsis |
| P9 | Normal | 0 | 57 | CHF |
| P10 | Obstructive | 0 | 55 | COPD |
| P11 | Obstructive | 1 | 75 | Postoperative |
| P12 | Obstructive | 1 | 72 | CHF |
| P13 | Obstructive | 1 | 79 | Postoperative |
| P14 | Obstructive | 0 | 62 | COPD |
| P15 | Obstructive | 1 | 29 | Pneumonia |
| P16 | Obstructive | 1 | 67 | COPD |
| **OVERALL** | **4/5/7** | **7 (43.8%)** | **55 (43-63)** | **/** |

Table 2S: Flow pattern, demographic data and etiology of respiratory failure.

ARDS: Acute Respiratory Distress Syndrome; CHF: Congestive Heart Failure; COPD: Chronic Obstructive Pulmonary Disease.

**Table 3S: Respiratory mechanics, major and minor asynchronies**

“Time const” refers to respiratory system time constant; “Autotriggered” refers to autotriggered (false positive) breaths; “Ineffective” refers to ineffective (false negative) efforts, i.e. patient breaths not detected by the ventilator; “Assisted” refers to assisted breaths, i.e. patient efforts detected and supported by the ventilator. Trigger delay, late cycling and early cycling were defined as machine ΔTi-start >0.25s, ΔTi-end >0.25s and ΔTi-end <-0.25s respectively. “Properly assisted” refers to patient breaths that are detected and supported by the ventilator without minor asynchronies, i.e. with both machine ΔTi-start and ΔTi-end <0.25s (absolute value).

| **Patient** | **Patient breaths**  **N** | **Detected breaths**  **N (%)** | **False positive**  **N (%)** | **False negative**  **N (%)** | **ΔTi-start**  **<0.25s**  **N (%)** | **ΔTi-end**  **<0.25s**  **N (%)** | **ΔTi-start/end**  **<0.25s**  **N (%)** |
| --- | --- | --- | --- | --- | --- | --- | --- |
| P1 | 280 | 280 (100.0) | 1 (0.4) | 0 (0.0) | 279 (99.6) | 279 (99.6) | 278 (99.3) |
| P2 | 230 | 230 (100.0) | 0 (0.0) | 0 (0.0) | 228 (99.1) | 230 (100.0) | 228 (99.1) |
| P3 | 311 | 311 (100.0) | 0 (0.0) | 0 (0.0) | 311 (100.0) | 309 (99.4) | 309 (99.4) |
| P4 | 268 | 268 (100.0) | 0 (0.0) | 0 (0.0) | 268 (100.0) | 268 (100.0) | 268 (100.0) |
| P5 | 131 | 131 (100.0) | 0 (0.0) | 0 (0.0) | 125 (95.4) | 130 (99.2) | 124 (94.7) |
| P6 | 572 | 571 (99.8) | 0 (0.0) | 1 (0.2) | 568 (99.5) | 517 (90.5) | 514 (90.0) |
| P7 | 215 | 215 (100.0) | 0 (0.0) | 0 (0.0) | 197 (91.6) | 214 (99.5) | 196 (91.2) |
| P8 | 230 | 229 (99.6) | 0 (0.0) | 1 (0.4) | 184 (80.3) | 229 (100.0) | 184 (80.3) |
| P9 | 455 | 444 (97.6) | 0 (0.0) | 11 (2.4) | 438 (98.6) | 381 (85.8) | 379 (85.4) |
| P10 | 457 | 450 (98.5) | 0 (0.0) | 7 (1.5) | 387 (86.0) | 445 (98.9) | 382 (84.9) |
| P11 | 162 | 162 (100.0) | 0 (0.0) | 0 (0.0) | 158 (97.5) | 162 (100.0) | 158 (97.5) |
| P12 | 215 | 215 (100.0) | 0 (0.0) | 0 (0.0) | 215 (100.0) | 214 (99.5) | 214 (99.5) |
| P13 | 178 | 178 (100.0) | 0 (0.0) | 0 (0.0) | 178 (100.0) | 178 (100.0) | 178 (100.0) |
| P14 | 399 | 398 (99.7) | 0 (0.0) | 1 (0.3) | 362 (91.0) | 394 (99.0) | 360 (90.5) |
| P15 | 83 | 83 (100.0) | 0 (0.0) | 0 (0.0) | 80 (96.4) | 83 (100.0) | 80 (96.4) |
| P16 | 234 | 232 (99.1) | 0 (0.0) | 2 (0.9) | 222 (95.7) | 229 (98.7) | 219 (94.4) |
| **OVERALL** | **4420** | **4397 (99.5)** | **1 (0.0)** | **23 (0.5)** | **4200 (95.5)** | **4262 (96.9)** | **4071 (92.6)** |

**Table 4S:** Performance of the waveform method in detecting patient spontaneous efforts.

Number (%) of efforts detected, false negative and false positive breaths are displayed in individual patients and in the overall population. Among detected efforts, number (%) of those with ΔTi-start <250ms, ΔTi-end <250ms and with both ΔTi-start <250ms and ΔTi-end <250ms (absolute values) are also displayed in individual patients and in the overall population.

|  |  | **Reference method** | | | |
| --- | --- | --- | --- | --- | --- |
|  |  | **auto trigger** | **assisted** | **ineffective** | **not**  **detected** |
| **Waveform method** | **Auto**  **Trigger** | 5 | 6 | 0 | 0 |
|  | **Assisted** | 1 | 3438 | 0 | 0 |
|  | **Ineffective** | 0 | 0 | 959 | 0 |
|  | **Not**  **Detected** | 0 | 0 | 17 | 0 |

**Table 5S: Agreement between waveform and reference assessment of major patient-ventilator asynchronies**

Breaths were assessed as assisted, ineffective, double-triggered or autotriggered with the waveform method (interpretation of standard ventilator waveforms) and the reference method (interpretation of standard ventilator waveforms and esophageal pressure). Global agreement between the reference and the waveform method was very good as assessed by a Cohen’s Kappa of 0.98 (0.98 – 0.99). No double-triggered breaths were observed. In the table “not detected” refers to breaths that were detected with one method, but not with the other one.
